# Reflectarray antennas: a smart solution for new generation satellite mega-constellations in space communications

**DOI:** 10.1038/s41598-020-78501-0

**Published:** 2020-12-09

**Authors:** Borja Imaz-Lueje, Daniel R. Prado, Manuel Arrebola, Marcos R. Pino

**Affiliations:** Department of Electrical Engineering, Group of Signal Theory and Communications, Universidad de Oviedo, Gijón, 33203 Spain

**Keywords:** Electrical and electronic engineering, Applied physics

## Abstract

One of the most ambitious projects in communications in recent years is the development of the so-called satellite mega-constellations. Comprised of hundreds or thousands of small and low-cost satellites, they aim to provide internet services in places without existing broadband access. For the antenna subsystem, reflectarrays have been proposed as a cheap solution due to their low profile and manufacturing costs, while still providing good performance. This paper presents a full design of a reflectarray antenna for mega-constellation satellites with a shaped-beam isoflux pattern for constant power flux in the surface of the Earth. A unit cell consisting of two stacked rectangular microstrip patches backed by a ground plane is employed, providing more than 360° of phase-shift. The generalized intersection approach optimization algorithm is employed to synthesize the required isoflux pattern in a 2 GHz bandwidth in Ku-band. To that purpose, a full-wave electromagnetic analysis is employed for the wideband design. The optimized reflectarray layout complies with the specifications of the isoflux pattern in the frequency band 16 GHz–18 GHz, demonstrating the capabilities of this type of antenna to provide a low-cost, low-profile solution for the user beam segment, including different types of shaped beams.

## Introduction

Since the Sputnik I, the first artificial satellite, was launched in October of 1957, the number of satellites orbiting the Earth has grown exponentially, serving a number of space missions including, but not limited to, television broadcast, mobile telephone networks, data transmission, remote sensing, radar and global positioning systems^1^. In this regard, one of the most ambitious projects in space communications in recent years is the development and deployment of satellite mega-constellations^2–4^, consisting of hundreds, or even thousands, of small satellites to provide worldwide broadband internet services with large bandwidth and low latency, even in remote places where there is no broadband access. Several companies such as Space X, OneWeb and Airbus have plans to develop these constellations^5^, which would require space debris mitigation plans^6,7^ due to the elevated risk of collisions^8,9^, and be cheap to manufacture^5^. Cube satellites, also known as CubeSats, have been gaining momentum in recent years for low earth orbit (LEO) missions. They provide a low-cost and low power consumption solution^10^, which makes them suitable for mega-constellation deployment. Since their invention in 1999 as an educational tool^11^, hundreds of CubeSats have been already been launched^12–14^ for missions including educational projects, communications, Earth observations, among others^1^. The Mars Cube One (MarCO), part of the NASA’s InSight mission to Mars, consisted of two 6U CubeSats known as MarCO-A and MarCO-B. They were the first-ever CubeSats to operate beyond the Earth’s orbit^15^.

The antenna subsystem is often one of the largest pieces of the satellite and traditionally it has been implemented by bulky parabolic reflectors or radiating phased-arrays^1^. In this regard, planar microstrip structures are interesting due to their low profile, mass and volume as well as their cheap manufacturing process^16^. In addition, it is easy to manipulate the radiation characteristics of these structures by tuning the geometrical features of their unit cell^17–20^. More specifically, reflectarray antennas^21^ are a type of spatially-fed planar arrays comprised of a planar structure of reflecting elements and a feed. Reflectarray antennas have already been proposed for space applications with very tight requirements, such as Direct Broadcast Satellite (DBS)^22–24^, Synthetic Radar Aperture (SAR)^25–27^ and global Earth coverage^28^. In addition, dual-polarized designs may be achieved by tuning two orthogonal geometrical features of the reflectarray element^29^. Large, wideband dual-polarized reflectarrays have been designed for space applications^30–32^, while smaller dual-polarized reflectarrays are usually designed to have pencil beam patterns^33–35^. Furthermore, due to their planar nature, deployable reflectarrays^36–39^ represent a good solution for high-gain antennas in CubeSats and other small satellites. In fact, some deployable reflectarrays have already been used in real missions. NASA’s ISARA mission was able to increase the CubeSat baseline downlink data rate from 9.6 kbps to 100 Mbps^40^ using a reflectarray comprised of three deployable panels on a 3U bus and achieving 33.5 dBi of gain at 26 GHz^41^. This mission put a reflectarray in space for the first time^42^. On the other hand, the two CubeSats of the MarCO mission also employ folded-panel reflectarrays, giving support to the 8.425 GHz Mars-to-Earth link with a gain of 29.2 dBi in right-hand circular polarization^43^. A deployable reflectarray for a potential follow-up of the RainCube mission has also been recently designed^44^. The European Space Agency is also considering reflectarrays for future CubeSat missions^45^. The M-ARGO deep-space spacecraft could be the first ESA mission for a deep-space CubeSat with a high gain reflectarray antenna^46^.

The considered reflectarrays for CubeSats missions are designed to focus the waves coming from the feed, achieving a highly directive beam pattern. This is very interesting for deep-space missions where distances are considerable^43^. However, for applications such as mega-constellations, having the satellites in LEO, it might be interesting to provide a different pattern, in particular, an isoflux pattern^28,47–51^ which distributes evenly the power radiated by the antenna over a portion of the Earth’s surface. Since for LEO there is a short time visibility of the satellite, this pattern would optimize the data downlink^50^. Thus, some kind of optimization procedure must be employed to obtain the phase law that provides the desired radiation characteristic from the antenna. In this regard, local search algorithms^52–54^ seem to be the choice for the optimization of very large reflectarrays for space applications.

In this paper, a low-cost, low-profile reflectarray antenna with a shaped-beam isoflux pattern is proposed for its use in small satellites for mega-constellations for the user beam in Ku band. A 2 GHz bandwidth of operation is considered, which is the same of the OneWeb service^55^ although at a different range, 16 GHz–18 GHz in the present case. The aim is to design a small reflectarray that can be easily accomodated in a small satellite and is able to provide service in a wideband with a shaped-beam for improved performance. The chosen unit cell consists of two stacked rectangular patches backed by a ground plane and it is able to provide more than 360° for the antenna design. This unit cell allows the designer to obtain a low-cost reflectarray antenna that works in dual-linear polarization. The near field of a standard gain horn is characterized by using a model based on spherical wave expansion and used through the reflectarray design process. A local search algorithm, namely the generalized intersection approach, is employed as optimization algorithm to seek the phase law that provides the desired radiation pattern at central frequency. After a layout has been obtained using a method of moments based on local periodicity (MoM-LP), it is optimized in a 2 GHz bandwidth to fulfil the isoflux pattern specifications. The results show that a good trade-off has been obtained between the relatively small size of the reflectarray antenna and the performance of the shaped-beam in the 2 GHz bandwidth.

## Analysis of reflectarray antennas

### Tangential field at the aperture

**Figure 1:**
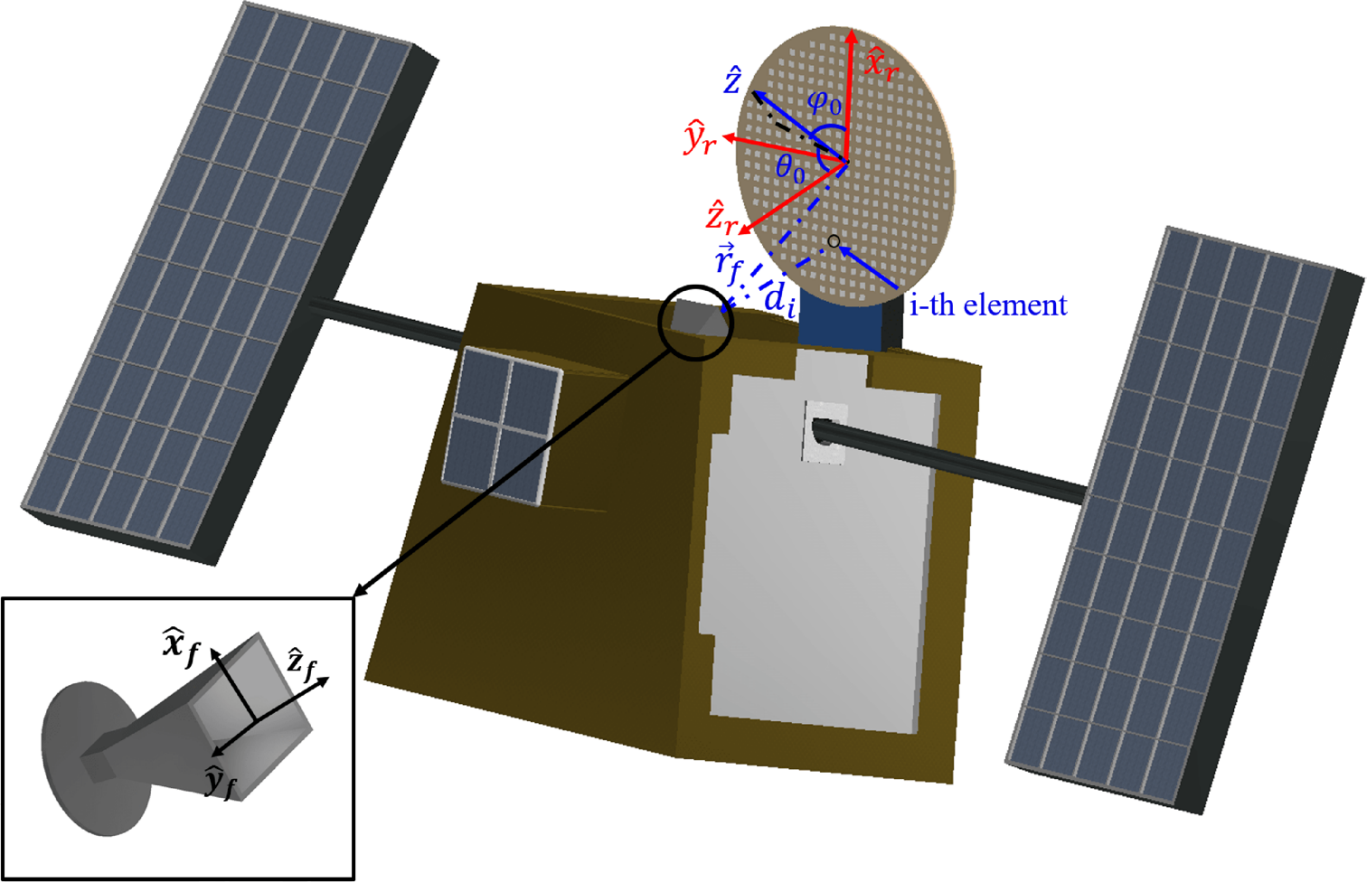
Sketch of the reflectarray mounted on a small satellite, including the diagram of the antenna optics under consideration. The reflectarray is elliptical and comprised of microstrip patches of variable size. The feed horn is placed in the XZ plane of the reflectarray coordinate system $(\hat{x}_r, \hat{y}_r, \hat{z}_r)$ and generates an incident field on its surface which is reflected back by the reflectarray.

A reflectarray antenna is comprised of a flat or slightly curved surface with reflective elements and a feed, as shown in Fig. 1. The figure shows that the reflectarray can be accommodated on the lateral side of the satellite for the launch and it can be easily deployed when it is installed in the desired orbit. The reflective elements are spatially fed and they are designed to adequately modify the impinging electromagnetic waves such that the radiated waves present the desired behaviour. This relation may be written as:

1 $$\begin{aligned} \vec {E}_{\mathrm{ref}}(\vec {r}_i) = \varvec{R}_i \ \vec {E}_{\mathrm{inc}}(\vec {r}_i), \end{aligned}$$

where $\vec {E}_{\mathrm{ref}}$ and $\vec {E}_{\mathrm{inc}}$ are the tangential reflected and incident fields at the reflectarray aperture, respectively; $\vec {r}_i=(x_i, y_i)$ are the coordinates of the *i*-th reflectarray unit cell and

2 $$\begin{aligned} \varvec{R}_i = \left( \begin{array}{cc} \rho _{xx,i} &{} \rho _{yx,i} \\ \rho _{yx,i} &{} \rho _{yy,i} \end{array} \right) \end{aligned}$$

is the matrix of reflection coefficients. This matrix fully characterizes the electromagnetic behaviour of the unit cell and it is calculated using a full-wave analysis tool assuming local periodicity, i.e., by embedding the unit cell in an infinite periodic environment comprised of the same cell. $\rho _{xx}$ and $\rho _{yy}$ are known as the direct coefficients, and control the shape of the copolar component of the radiation pattern through their phase and losses through their magnitude. On the other hand, $\rho _{xy}$ and $\rho _{yx}$ are known as cross-coefficient and constitute an important source of cross-polarization. Once the tangential reflected electric field has been obtained using Eq. (1), the magnetic field may be readily calculated:

3 $$\begin{aligned} \vec {H}_{\mathrm{ref}}(\vec {r}_i) = \dfrac{\vec {k}_{\mathrm{ref}} \times \vec {E}_{\mathrm{ref}}(\vec {r}_i)}{2\pi f_0 \mu _0}, \end{aligned}$$

where $\mu _0$ is the permeability of vacuum and:

4 $$\begin{aligned} \vec {k}_{\mathrm{ref}} = -k_0\sin \theta _i\cos \varphi _i\hat{x} - k_0\sin \theta _i\sin \varphi _i\hat{y} + k_0\cos \theta _i\hat{z}, \end{aligned}$$

where $(\theta _i, \varphi _i)$ is the incident angle of the plane wave for the *i*-th element, and $\vec {k}_{\mathrm{ref}}$ corresponds to the reflected propagative wave in the specular direction in the absence of grating lobes^53^. For the cross product in Eq. (3), the $E_z$-component of the reflected electric field is needed. It can be obtained by means of the plane wave relation $\vec {k}_{\mathrm{ref}}\cdot \vec {E}_{\mathrm{ref}}=0$.

### Computation of the far field

Once the tangential fields at the aperture, both electric and magnetic, have been obtained, the radiation pattern may be readily computed. According to the first principle of equivalence in electromagnetics, the far field radiated by an aperture antenna is $\vec {E} = E_{\theta }\hat{\theta }+ E_{\varphi }\hat{\varphi }$, where the components take the following form^56^:

5 $$\begin{aligned} E_{\theta }&= \dfrac{jk_0\exp \left( -jk_0r\right) }{4\pi r} \left[ P_x \cos \varphi + P_y \sin \varphi - \eta _0\cos \theta \left( Q_x \sin \varphi - Q_y \cos \varphi \right) \right] , \end{aligned}$$

6 $$\begin{aligned} E_{\varphi }&=-\dfrac{jk_0\exp \left( -jk_0r\right) }{4\pi r} \left[ \eta _0 \left( Q_x \cos \varphi +Q_y \sin \varphi \right) + \cos \theta \left( P_x \sin \varphi - P_y \sin \varphi \right) \right] , \end{aligned}$$

where $\eta _0 = \mu _0 c_0$ is the vacuum impedance and $P_{x/y}$ and $Q_{x/y}$ are the spectrum functions, which can be calculated as the Fourier transforms of the tangential electric and magnetic fields in the aperture, respectively^57^. After a few manipulations^21^, the spectrum functions may be expressed as a 2-D inverse fast Fourier transform, which may be efficiently computed using the FFT algorithm. Then, the copolar (desired) and cross-polar (undesired) component of the far field are obtained for both linear polarizations. For the case of polarization X, they are obtained as:

7 $$\begin{aligned} \left( \begin{array}{l} E_{\mathrm{cp}}^X \\ E_{\mathrm{xp}}^X \end{array} \right) =\left( \begin{array}{ll} \,\cos \varphi &{} -\sin \varphi \\ -\sin \varphi &{} -\cos \varphi \end{array} \right) \left( \begin{array}{l} E_{\theta }^X \\ E_{\varphi }^X \end{array} \right) , \end{aligned}$$

while for polarization Y they are:

8 $$\begin{aligned} \left( \begin{array}{l} E_{\mathrm{cp}}^Y \\ E_{\mathrm{xp}}^Y \end{array} \right) = \left( \begin{array}{ll} \sin \varphi &{} \,\cos \varphi \\ \cos \varphi &{} -\sin \varphi \end{array} \right) \left( \begin{array}{l} E_{\theta }^Y \\ E_{\varphi }^Y \end{array} \right) . \end{aligned}$$

For the purposes of this work, we will only consider the copolar component of the far field, $E_{\mathrm{cp}}$.

## Optimization algorithm

### Analytical solution for a collimated beam

According to the well established array theory, there is an analytical solution to obtain a collimated beam at an arbitrary direction $(\theta _0,\varphi _0)$. In such case, the phase-shift introduced by each unit cell is^21^:

9 $$\begin{aligned} \angle \rho _{xx,i} = \angle \rho _{yy,i} =k_0 \left( d_i - \left( x_i\cos \varphi _o + y_i\sin \varphi _o\right) \sin \theta _0\right) , \end{aligned}$$

where $d_i$ is the distance from the phase-center of the feed to the *i*-th element placed at $\vec {r}_i=(x_i, y_i)$ in the reflectarray coordinate system (see Fig. 1). The phase-shift in (9) corresponds to the phase of one of the direct reflection coefficients in Eq. (2). For the reflectarray design, the reflection coefficient phase is adjusted to match the required phase-shift. This is done by tuning one or more geometrical features of the reflectarray unit cell, for instance, the length of a dipole. If the unit cell is able to work in dual polarization, the phases of $\rho _{xx}$ and $\rho _{yy}$ may be independently adjusted. A classical example is the rectangular patch, which can match each phase of the direct reflection coefficients by tuning both dimensions of the patch^29^.

The collimated beam obtained by implementing the phases of Eq. (9) is useful for applications such as point-to-point communications. However, other applications require more complex shaped beams, which in general may not be obtained by analytical equations. In such cases, an optimization algorithm is used to synthesize the required phase-shift, and the initial phases provided by Eq. (9) may be used as starting point for the optimization.

**Figure 2:**
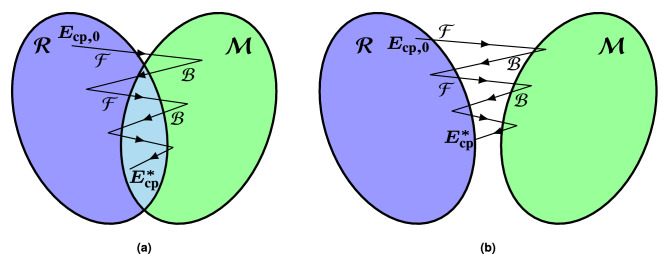
Schematic representation of the intersection approach optimization algorithm. This algorithm is iterative and considers two sets: the set of far fields that can be radiated by the antenna (${\mathcal {R}}$), and the set of far fields that comply with the specifications (${\mathcal {M}}$). Starting with an initial guess ($E_{\mathrm{cp},0}$), the algorithm applies two successive operations at each iteration: the forward projection (${\mathcal {F}}$) and the backward projection (${\mathcal {B}}$) until a solution $E_{\mathrm{cp}}^*$ is reached if the two sets intersect (**a**), or the minimum distance if the intersection is void (**b**).

### The generalized intersection approach

For this work, we have chosen the generalized Intersection Approach (gIA)^58^ as optimization algorithm. It is an iterative algorithm which works with two sets: the set of far fields that can be radiated by the reflectarray antenna (set ${\mathcal {R}}$), and the set of radiation patterns that comply with the requirements (set ${\mathcal {M}}$). At each iteration *n*, the gIA performs the following operation:

10 $$\begin{aligned} E_{\mathrm{cp}, n+1} = {\mathcal {B}}\left[ {\mathcal {F}} \left( E_{\mathrm{cp},n} \right) \right] , \end{aligned}$$

where ${\mathcal {F}}$ is known as the forward projection and ${\mathcal {B}}$ is the backward projection. The forward projection projects $E_{\mathrm{cp}}$ onto the set of far fields that comply with the specifications, while the backward projection projects the far field which fulfils specifications onto the set of far fields which can be radiated by the antenna. The aim of the sequence defined in Eq. (10) is to find the intersection between the two sets, or if that is not possible because the intersection is void, to find a far field belonging to set ${\mathcal {R}}$ whose distance to the set ${\mathcal {M}}$ is minimal. Figure 2 shows a schematic representation of this process. In addition, the gIA is a local optimizer, and thus the starting point for the optimization is of great importance, since it will determine the quality of the obtained results. Nevertheless, it has been shown that starting with a properly focused reflectarray, whose phase-shift is given by Eq. (9), is a good enough starting point for a general optimization procedure^59^.

### The forward projection

**Figure 3:**
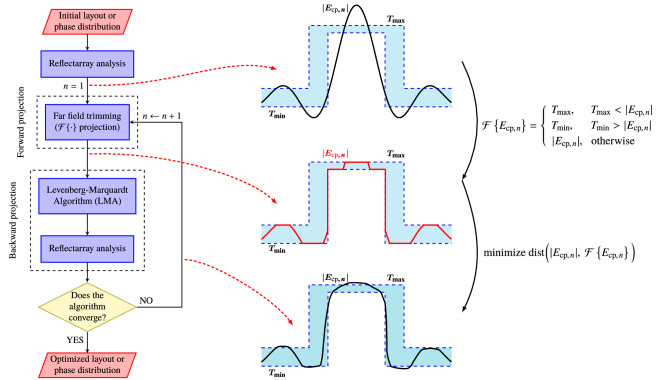
Flowchart of the generalized intersection approach optimization algorithm particularized for reflectarray antennas and a copolar-only synthesis. After the reflectarray analysis, the radiation pattern is obtained. Then, it is trimmed by the forward projection, which imposes the specifications as a set of minimum ($T_{\min }$) and maximum ($T_{\max }$) templates or masks. Then, the backward projector minimizes the distance between the current far field and the trimmed far field using the Levenberg-Marquardt algorithm. This process is repeated until the algorithm converges.

The computational efficiency and convergence properties of the algorithm largely depend on the implementation of the two projectors. The first step is the definition of the forward projector, which imposes the restrictions on the radiated field. They are given in the form of far field masks. If $T_{\min }$ and $T_{\max }$ are the minimum and maximum specification masks that the far field must fulfil, they are imposed as shown in Fig. 3. In this way, the result of the forward projection is a far field that complies with the requirements but that in general cannot be radiated by the reflectarray. Since the forward projection only imposes the requirements in the far field through masks, its computational cost is negligible.

### The backward projector

The goal of the backward projector is to decrease the distance between the current far field radiated by the reflectarray and the far field obtained by the forward projector that complies with the specifications (see Fig. 3). For this purpose, a general optimization algorithm is employed, the Levenberg–Marquardt algorithm (LMA)^60^ in the present case. In addition, there is no need to find a minimum^58^ (in general, local) in this step, only to decrease the distance at each iteration of the intersection approach. Thus, only a few iterations of the LMA are needed, and in the present case, they are set to three. Further details concerning the algorithm may be found in other works^53,60^. Finally, the radiation pattern is computed with the updated solution of the LMA.

It is worth noting that, even though a general optimization algorithm is employed in the backward projector, it is only employed to decrease a distance between two far fields (see Fig. 3), not to attain a minimum (in general local). In this regard, it is the gIA the algorithm responsible for seeking the desired solution by iteratively applying the forward projector first and then the backward projector, as in Eq. (10).

## Antenna design

### Application requirements

**Figure 4:**
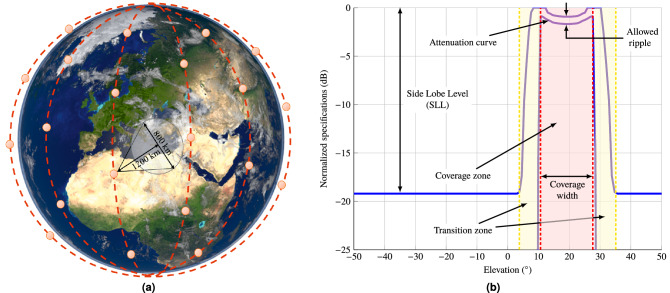
(**a**) Illustration of the specific requirements for orbit and coverage size (not to scale). The selected orbit is LEO at 1200 km above ground and the spot size is 800 km long in diameter. From these values, the isoflux pattern can be generated. (**b**) General isoflux pattern specifications. The isoflux pattern is specified by the attenuation curve computed using the Friis equation and depends on the working frequency and distance from the antenna to each point in the surface of the Earth over a given coverage width. The specifications also include an allowable ripple, a transition zone and side lobe level.

The general isoflux pattern specification is shown in Fig. 4. It consists of a coverage zone, transition zone and non-coverage zone. The coverage zone is defined by the attenuation curve, coverage width and the allowed ripple. The attenuation curve is given by the Friis equation^61^ and depends on the working frequency and the distance of the antenna to the surface of the Earth. Details on its calculation may be found elsewhere^28^. The coverage width depends on the portion of the Earth surface which will be covered by the antenna providing constant power flux. The transition zone is provided to facilitate convergence of the optimization algorithm while imposing a rapid transition to the non-coverage zone, which is defined by the maximum side lobe level.

For this work, the antenna will be mounted in a small satellite in LEO at 1200 km above the surface of the Earth, considering a circular beam footprint with a radius of 400 km. This gives a coverage width of $36.5^{\circ }$. In addition, the allowed ripple and side lobe level are set to 2 dB and 15 dB, respectively. Finally, the working frequency is $f_0 = 17\,\hbox {GHz}$ and we will consider a bandwidth of 2 GHz, from 16 to 18 GHz, which is an 11% bandwidth. These requirements were selected as a compromise between the size of the antenna (see “Antenna structure” section) and the expected performance. Better performance could be achieved in terms of ripple, side lobe level, etc. if a larger antenna size is considered.

### Unit cell characterization

**Figure 5:**
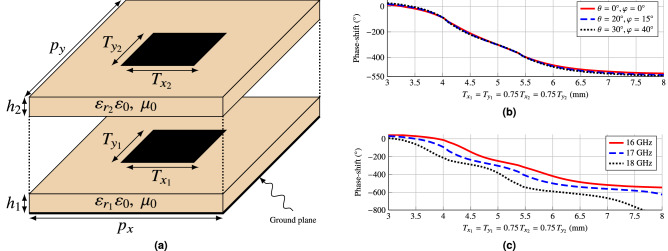
(**a**) Sketch of the employed unit cell. It consists in two stacked rectangular patches (here drawn separately) backed by a ground plane with periodicity $p_x=p_y=8.82\,\hbox {mm}$. The commercial substrate Rogers 3003 has been employed for both layers with thickness of $h_1=30\,\hbox {mil}$ for the bottom layer and $h_2=60\,\hbox {mil}$ for the top layer. (**b**) Phase response of the unit cell as a function of the patch size for different angles of incidence at 17 GHz. As it can be seen, the phase-shift is stable for all considered angles. (**c**) Phase-shift introduced by the unit cell for different frequencies for oblique incidence with ($\theta ,\varphi )=(40^{\circ }, 30^{\circ }$).

The chosen unit cell consists in two stacked rectangular patches backed by a ground plane, as shown in Fig. 5a. Since the unit cell has two layers, it will be able to provide more than a full phase shift range of 360°. For the substrate, the commercially available Rogers 3003 has been chosen for both layers, with a dielectric constant of $\varepsilon _r=3$ and a loss tangent of $\tan \delta =0.001$ at 10 GHz. A thickness of $h_1=0.762\,\hbox {mm}$ (30 mil) has been selected for the bottom layer, while the top layer has a thickness of $h_2=1.524\,\hbox {mm}$ (60 mil). Figure 5b shows the response of the unit cell for different angles of incidence at 17 GHz. It is shown that the angle of incidence barely affects the phase-shift produced by the unit cell, demonstrating its angular stability. On the other hand, Fig. 5c shows the response for several frequencies for an oblique incidence of ($\theta ,\varphi )=(40^{\circ }, 30^{\circ }$). The unit cell is able to provide more than $500^{\circ }$ of phase-shift for different frequencies and angles of incidence, which makes it suitable for a wideband optimization procedure. This would not be possible with a single layer of rectangular patches due to the limited phase-shift range^21^.

**Figure 6:**
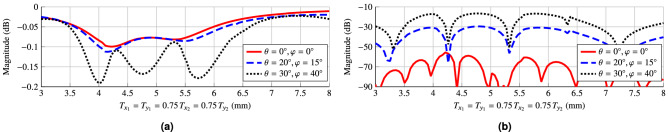
Magnitude response of the unit cell at 17 GHz for different angles of incidence for the (**a**) direct coefficient and (**b**) cross-coefficient. The losses remain low and stable for low oblique incidence while the cross-polarization increases with the angle of incidence.

The magnitude response of the unit cell is shown in Fig. 6, for the direct coefficient (Fig. 6a) and the cross-coefficient (Fig. 6b) at 17 GHz. The losses remain low and stable for low angles of incidence. In fact, for $\theta =20^{\circ }$ the maximum losses are around 0.1 dB and they increase for higher angles of incidence, but remain below 0.2 dB. On the other hand, the cross-polarization, shown in Fig. 6b, is very low for normal incidence and rapidly increases with the angle of incidence.

### Antenna structure

The architecture of the antenna is shown in Fig. 1. It is a circular reflectarray in single-offset configuration with a horn as feed. The reflectarray is comprised of 366 unit cells in a regular grid of $22\times 21$ elements in the main axes. The periodicity is $0.5\lambda _0$ at the working frequency ($f_0 = 17\,\hbox {GHz}$). In addition, a dielectric frame is considered to screw the reflectarray to the supporting structure, with a width of $0.5\lambda _0$. Thus, the total dimensions of the reflectarray in its main axes are $203\,\hbox {mm}\times 194\,\hbox {mm}$. For the feed, the Narda 639 standard gain horn has been selected. Its near field has been characterized by means of full-wave simulations and a model based on spherical wave expansion to obtain the field at the reflectarray aperture. This model will be used throughout the design process with the exception of the first stage of synthesis, where a $\cos ^q\theta$ model is employed^62^, adjusting the *q* parameter to match the main lobe of the horn far field. The center of the horn aperture is placed at $\vec {r}_f =(-79, 0, 164)\,\hbox {mm}$ in the reflectarray coordinate system (see Fig. 1). The illumination angle is $22.5^{\circ }$ while the radiation angle is $\theta _0=23^{\circ }$. These values have been chosen to satisfy three conditions, namely, avoid feed blockage, achieve a concentric phase-shift for the initial reflectarray design using Eq. (9), and to provide an illumination taper around −14 dB. Specifically, the illumination taper varies with frequency, from $-13.6\,\hbox {dB}$ at 16 GHz to $-14.2\,\hbox {dB}$ at 18 GHz.

### Results

**Figure 7:**
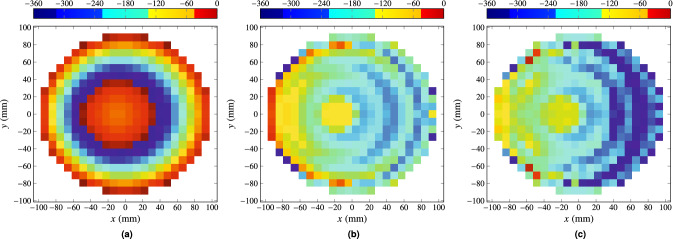
Required phase-shift at each reflectarray element to (**a**) achieve a pencil beam pattern pointing at ($\theta _0, \varphi _0)=(23^{\circ }, 0^{\circ }$); (**b**) obtain an isoflux pattern using an ideal far field model for the feed horn based on a $\cos ^q\theta$ function with $q=15$; and (**c**) obtain an isoflux pattern using the spherical wave expansion model for the feed horn. Figure created with PGFPlots^63^.

**Figure 8:**
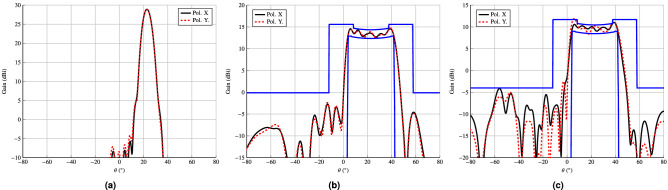
Radiation patterns corresponding to the phase-shift distributions shown in Fig. 7. (a) Pencil beam pointing at ($\theta _0, \varphi _0)=(23^{\circ }, 0^{\circ }$). (**b**) Isoflux pattern obtained using an ideal $\cos ^q\theta$ model for the feed horn. (**c**) Isoflux pattern obtained using the spherical wave expansion model for the feed horn.

The first step in the design process is a Phase-Only Synthesis (POS). To that end, the gIA is employed (see Fig. 3). For the POS, the initial phase distribution is given by Eq. (9), setting ($\theta _0, \varphi _0)=(23^{\circ }, 0^{\circ }$), to obtain a pencil beam focused in the direction of the desired isoflux pattern. This initial phase-shift distribution is shown in Fig. 7a and generates the radiation pattern shown in Fig. 8a. For the first stage of POS, an ideal far field model is employed for the feed horn, based on the $\cos ^q\theta$ model with $q=15$. The resulting phase-shift distribution is shown in Fig. 7b and the generated far field in Fig. 8b. This radiation pattern complies with the specifications in terms of coverage width, ripple and side lobes. However, since and ideal feed model was used, another stage of synthesis is required, this time using the spherical wave expansion model. The final phase-shift distribution is shown in Fig. 7c. It is very similar to the phase-shift obtained in the previous stage since the starting point for this optimization was close to a solution. Figure 8c shows the far field for this last stage and it also complies with the requirements. The phase-shift distributions shown in Fig. 7 correspond to linear polarization X, and similar results were obtained for polarization Y.

Once the desired phase-shift distribution has been synthesized, the following step is to obtain a reflectarray layout. With that goal, a method of moments based on local periodicity (MoM-LP)^64^ will be employed to characterize the electromagnetic behaviour of the unit cell. The layout is obtained by adjusting, element by element, the size of the patches to match the required phase-shift. For this task, the patch dimensions of both layers are fixed with $T_x = T_{x_1} = 0.75T_{x_2}$ and $T_y =T_{y_1} = 0.75T_{y_2}$ [see Fig. 5a]. Then, $T_x$ and $T_y$ are swept independently to match the required phase-shift for each linear polarization. Finally, both variables are adjusted simultaneously to take into account the coupling between both polarizations.

**Figure 9:**
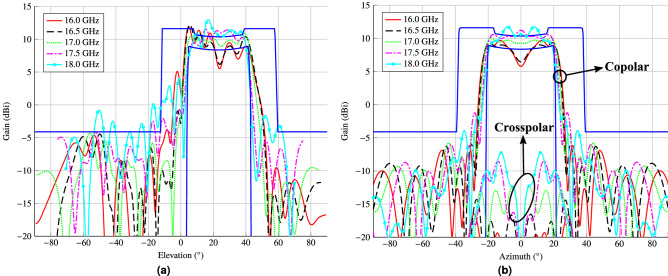
Main cuts in (**a**) elevation and (**b**) azimuth of the reflectarray layout design obtained after the phase-only synthesis. This design complies with specifications at central frequency (17 GHz), but due to its narrowband nature, it does not comply in the rest of the band. Due to the symmetry in the antenna optics, there is a null in the crosspolar pattern in the elevation cut.

**Figure 10:**
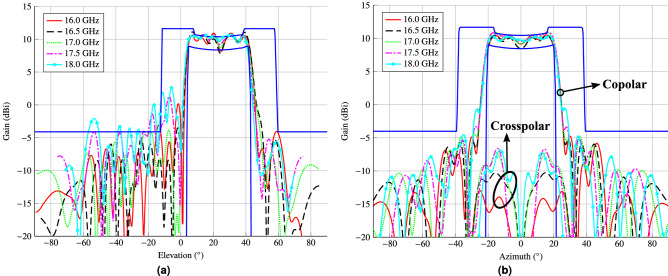
Main cuts in (**a**) elevation and (**b**) azimuth of the design after the wideband copolar-only optimization. This design almost fulfils specifications in a 2 GHz bandwidth (11%), achieving a good trade-off between performance of a wideband shaped-beam reflectarray and its physical size. Due to the symmetry in the antenna optics, there is a null in the crosspolar pattern in the elevation cut.

The reflectarray obtained in this way is narrowband, since the POS and layout design process only took into account the electromagnetic behaviour of the unit cell at a single frequency. Thus, outside a narrow band around the design frequency, the reflectarray will not comply with the requirements. This is verified in the main cuts in elevation and azimuth that are shown in Fig. 9 for the frequency range 16 GHz–18 GHz. They were obtained by simulating the initial layout with the full-wave MoM-LP tool and the spherical wave expansion model for the feed horn. As it can be seen, only at central frequency does the reflectarray comply with the mask requirements. At other frequencies there is a considerable ripple in the coverage zone and at the extreme upper frequency the secondary lobes are high.

**Figure 11:**
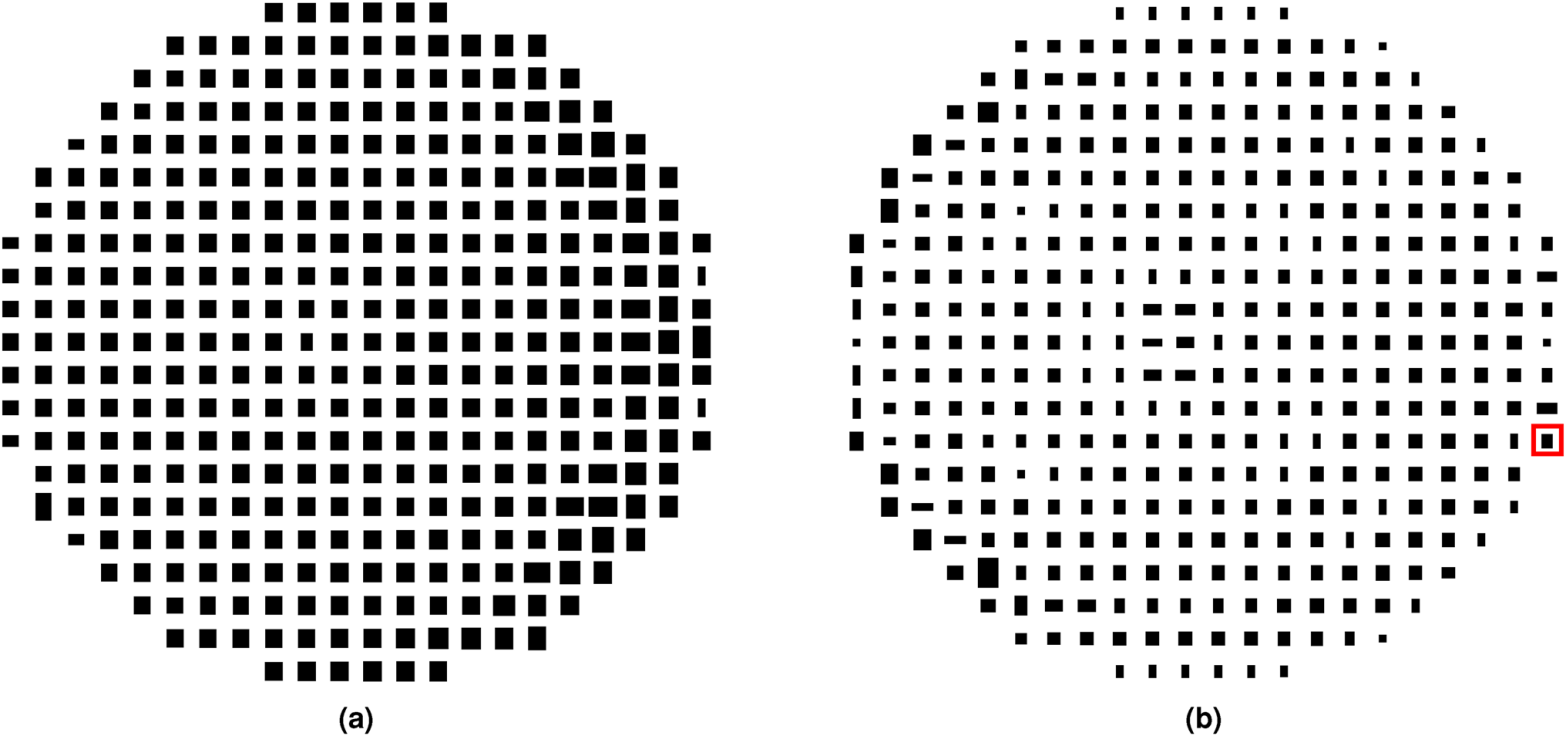
Layout of the shaped-beam reflectarray optimized in a 2 GHz bandwidth with an isoflux pattern. (**a**) Bottom layer. (**b**) Top layer. The dimensions of the highlighted patch are $2.952\,\hbox {mm}\times 3.652\,\hbox {mm}$.

**Figure 12:**
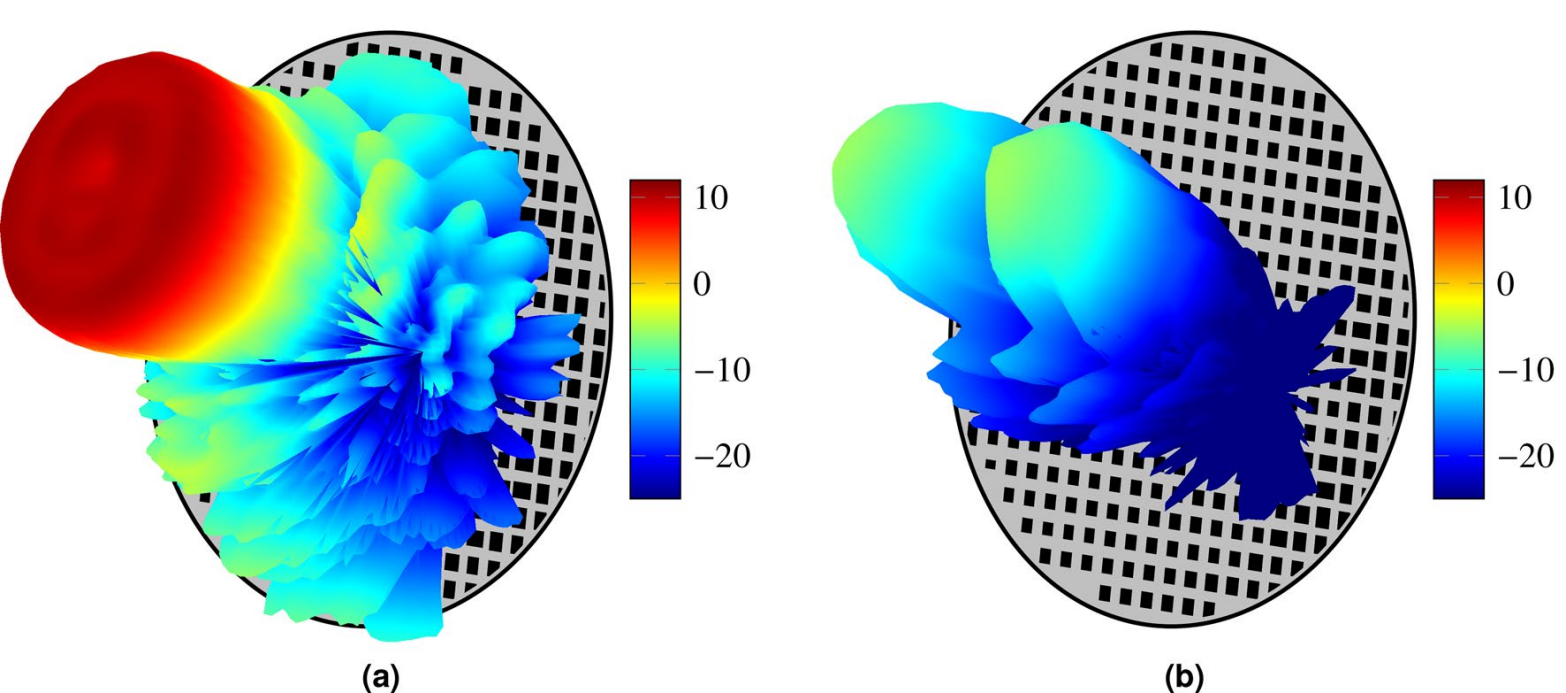
3D representation of the (**a**) copolar and (**b**) crosspolar patterns for polarization X.

In order to improve the bandwidth of the reflectarray antenna, an optimization at several frequencies must be carried out. To that end, the same algorithm is employed, i.e., the gIA whose flowchart is shown in Fig. 3. However, this time the optimizing variables will be the dimensions of the patches for both layers, instead of the phase-shift introduced by the elements as it was the case for the POS. In addition, for the optimization in a certain bandwidth we need to take into account the behaviour at different frequencies of the unit cell. Thus, the MoM-LP tool is employed directly in the optimization loop to perform a copolar wideband design. It is carried out at five equidistant frequencies in the range 16 GHz–18 GHz and the same specification masks are imposed in all of them. The wideband design is divided into three steps to accelerate the process and improve convergence towards a solution. First, only the patterns for polarization X are optimized, by only considering the $T_{x_1}$ and $T_{x_2}$ variables for all the reflectarray elements. Then, the same is applied for polarization Y, with variables $T_{y_1}$ and $T_{y_2}$. Finally, both polarizations are optimized at the same time considering four optimizing variables per element. This is done in order to take into account the coupling between polarizations. The result of the wideband design is a net improvement at frequencies other than 17 GHz at which the initial design was performed, as shown in Fig. 10. Now, the optimized reflectarray practically fulfils the specifications for the coverage area and side lobes in a 2 GHz bandwidth. In fact, only at the upper frequency are the side lobes slightly higher than the mask. Nevertheless, they were also reduced by the wideband design. Figure 11 shows the corresponding optimized layout.

Finally, Fig. 12 shows a 3D representation of the isoflux pattern radiated by the antenna. Here, it is appreciated the circular ring that provides the spot with constant power flux. The results presented in Figs. 9, 10 and 12 are for polarization X. However, similar results were also achieved for polarization Y, obtaining a small reflectarray working in dual-linear polarization suitable for the new generation of small-satellite mega-constellations, which will be able to provide data connections worldwide. It is worth noting that the methodology presented in this work may be readily extended to circular polarization with the current unit cell or to dual-circular polarization provided an appropriate reflectarray unit cell.

## Discussion

This work has presented the design of a small, low-cost, low-profile reflectarray antenna for its use in small satellites for mega-constellations. One feature of the design is to provide a shaped beam with an isoflux pattern to achieve constant flux over a wide region, since it allows to optimize the data downlink. In addition, the antenna has been designed to work in the Ku band with a bandwidth of 2 GHz, aiming for the user beam segment. The specifications for the isoflux pattern are a ripple of the coverage zone of 2 dB and a side lobe level of 15 dB. These requirements will be fulfilled by a small reflectarray comprised of 366 unit cells an a size of approximately $200\,\hbox {mm}^{2}$. In order to comply with the 2 GHz bandwidth with a shaped beam, a unit cell consisting in two stacked rectangular patches is used. This unit cell has been proven to have good angular stability and to provide enough phase-shift and low losses for a wideband design. The design process is based on the use of the generalized intersection approach, a powerful algorithm for antenna synthesis. The procedure is divided in two stages. First, a design at central frequency is carried out, obtaining a narrowband layout which presents a high ripple in most of the 2 GHz bandwidth. Then, a wideband design is performed to greatly improve the performance of the antenna. The optimized layout fulfils with the specifications in the whole bandwidth, presenting slightly high side lobe level at the upper frequency.

Compared to other dual-polarized reflectarrays in the literature, dual-polarized shaped-beam reflectarrays have been designed for space applications in geostationary orbit^30–32^. These are very large reflectarrays with high gain, unsuitable for small satellites. On the other hand, previously proposed smaller dual-polarized reflectarrays suitable for SmallSats have directive pencil beams^33–35^. However, reflectarrays offer the possibility of an optimized coverage with regard to directive pencil beams, by enlarging the coverage area and providing a constant power flux. In the case of SmallSats for mega-constellations, the proposed antenna is small, light-weight, compact and easy to deploy. In fact, the thickness of the reflectarray panel is only 2.3 mm, and since it is planar, it has very low volume which is easy to stow on the side of a SmallSat for its subsequent deployment. At the same time, we improve the coverage by synthesizing an isoflux pattern. Moreover, the designed reflectarray is based on PCB (printed circuit board), a very well-known and mature technology that enables rapid mass-production at low-cost, which is ideal for mega-constellations of small satellites.

The results shown in this work demonstrate the capability of reflectarray antennas to offer a cheap solution while providing good performance for small satellites in mega-constellations. At the same time, the design has been carried out with a trade-off of reflectarray physical size and performance, obtaining a small antenna with a shaped-beam that almost fulfils requirements in an 11% bandwidth. In this regard, better performance regarding ripple, side lobes, bandwidth and gain could be achieved by increasing the size of the antenna. Finally, although the design was performed in dual-linear polarization, the design procedure is general and could be applied to circular or dual-circular polarization provided a suitable reflectarray element.

## Methods

In-house tools were developed to implement the POS and wideband design algorithms based on the generalized intersection approach (gIA) and the full-wave method of moments based on local periodicity (MoM-LP). Details about aspects of the implementation of the gIA may be consulted elsewhere^53,60^. The MoM-LP formulation is based on the generalized scattering matrix in the spectral domain^64^, and the implemented tool has been widely validated through full-wave simulations and prototypes^21^.
